# Primary anterior perineal hernia: A case report and review of the literature

**DOI:** 10.1111/ases.12800

**Published:** 2020-04-01

**Authors:** Ikuo Watanobe, Shozo Miyano, Michio Machida, Hiroyuki Sugo

**Affiliations:** ^1^ Juntendo University Nerima Hospital Tokyo Japan

**Keywords:** laparoscopic repair, primary anterior perineal hernia, primary perineal hernia

## Abstract

Perineal hernia is a type of pelvic floor hernia and an extremely rare pathologic state. Perineal hernias can be classified into anterior and posterior types according to their positional relationship to the superficial transverse perineal muscle. A 49‐year‐old woman presented with bulging of the right labium major while standing. Standing external ultrasonography revealed a mass in the bulge, which could not be identified by transvaginal ultrasonography, CT, or MRI. Although hernia content could not be identified preoperatively, the patient was given a diagnosis of primary perineal hernia and underwent laparoscopic repair. Symptoms resolved postoperatively, and no sign of relapse has been noted for 8 months postoperatively. Here, we report the case details and review previous case reports.

## 1 INTRODUCTION

Pelvic floor hernia is extremely rare, particularly obturator, perineal, and sciatic hernias.^1^ Primary perineal hernias occur most commonly in individuals aged 40 to 60 years and are five times more common in women than in men.^1^ Controversy remains regarding treatment, with various surgical approaches and hernia repair techniques reported in the literature. This appears to be due to varying sites and sizes of hernial orifices and the anatomical complexity of the pelvic floor. Therefore, it currently appears to be necessary to individualize treatment strategies for each case.

## 2 CASE PRESENTATION

For 5 months before presentation, a 49‐year‐old woman (BMI, 18.7 kg/m^2^) noticed pudendal discomfort and swelling that occurred after prolonged standing. She consulted our hospital because the discomfort had recently progressed to pain while standing. On presentation, a thumb tip‐sized mass was seen in the right labium major on standing and disappeared on lying down. There was no tenderness, but she had mild pain at the site when increased intra‐abdominal pressure occurred such as during defecation. She had never been pregnant. Her medical history included an 8‐cm uterine myoma diagnosed at age 45 years for which she was being observed.

**Figure 1:**
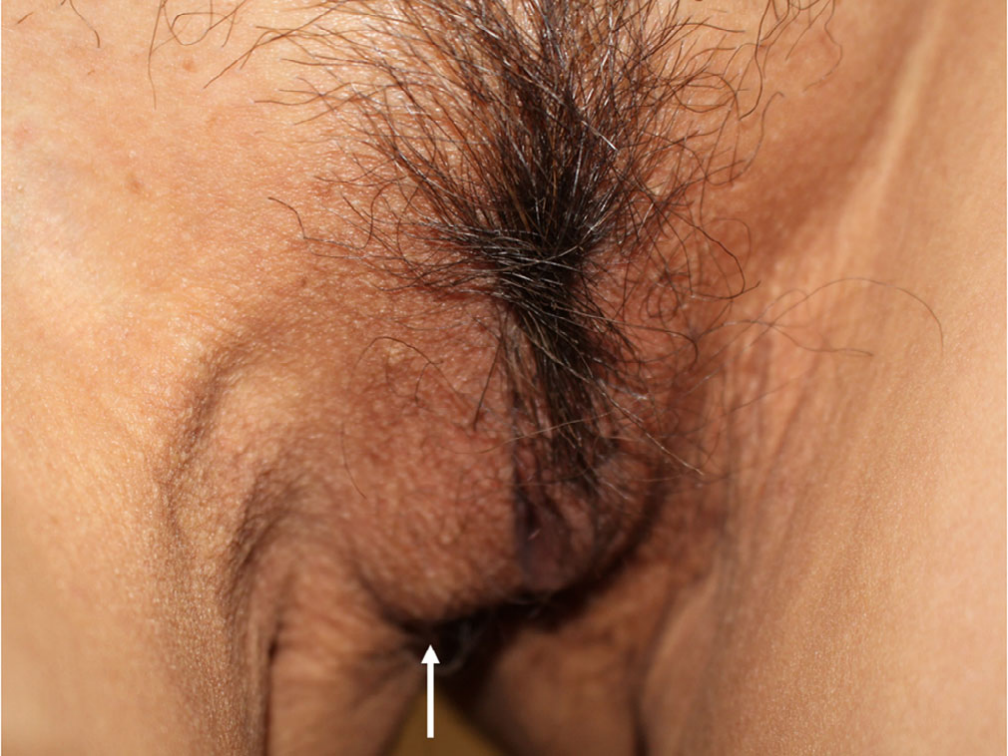
Perineal region of the patient in the standing position. A bulging mass (arrow) noted in the right labium major in the standing position. A mobile, relatively soft mass was palpable directly under the skin

**Figure 2:**
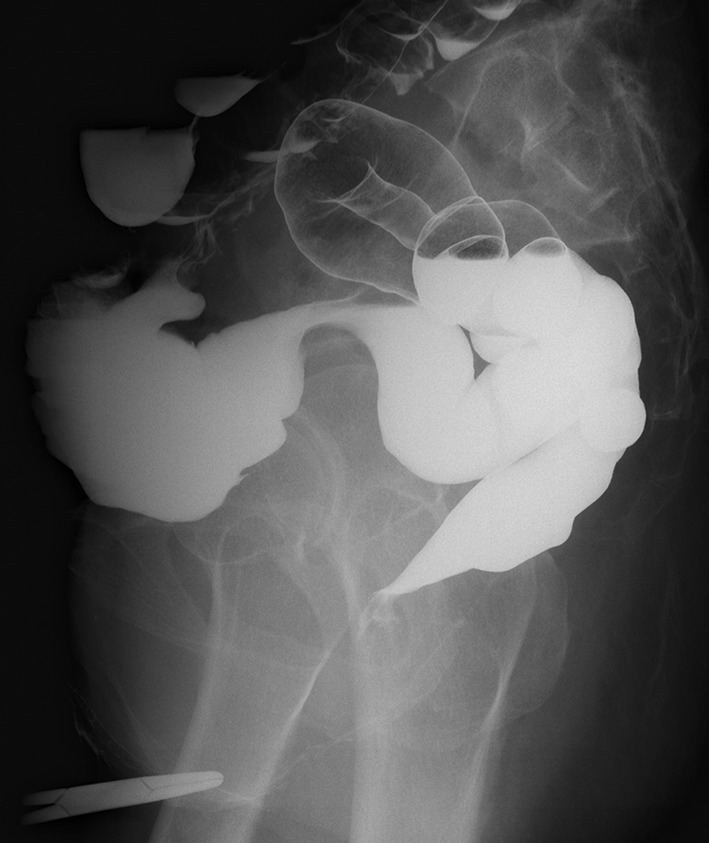
Barium enema image (taken while the patient was standing). The mass observed in the perineal region is indicated by the tip of the forceps. The possibility of the hernia content being rectum or colon was excluded

When the patient was in a standing position, bulging of the right labium major was noted with a palpable mass (Figure 1). Standing external ultrasonography revealed a 3‐cm oval, partially hypoechoic mass. Serial ultrasonography showed no intestinal peristalsis. Transvaginal ultrasonography revealed no corresponding mass in the pelvic floor region. CT and MRI showed no findings suggestive of a hernia, atrophy, a pelvic floor musculature defect, or a mass shadow in the perineal region. Barium enema showed no intestine reaching the pelvic floor, and no intestinal prolapse was observed on images taken with the patient in the standing position with abdominal pressure (Figure 2).

**Figure 3:**
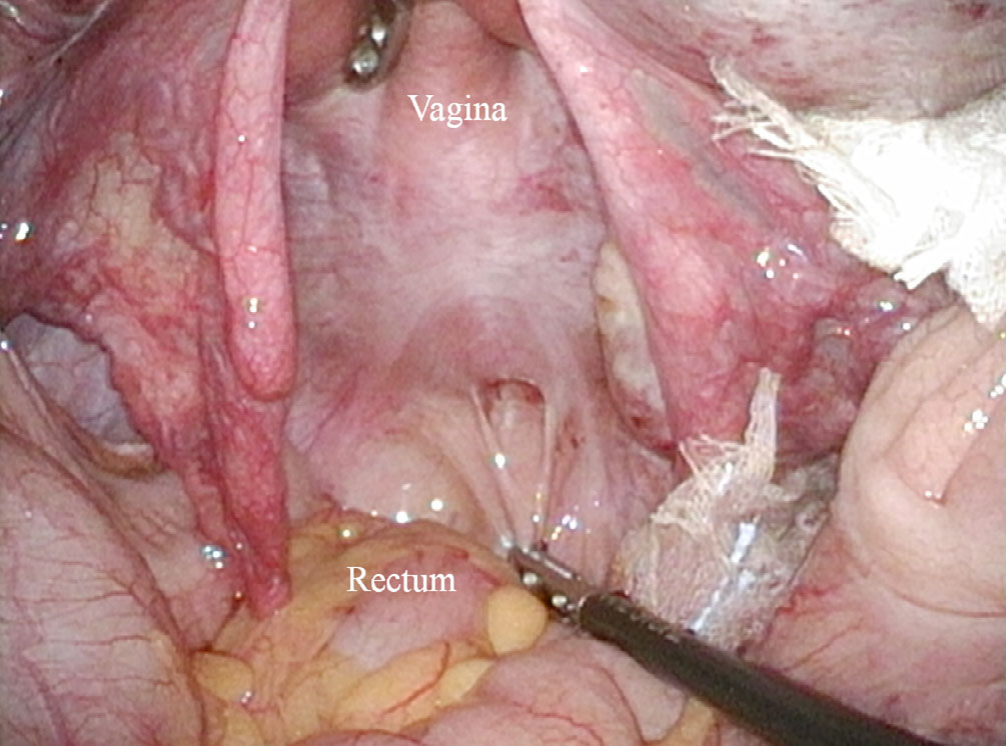
Hernial orifice found intraoperatively. A 2‐cm hernial orifice was found in the pouch of Douglas; the lumen extended up to the perineal region

Although the hernia content could not be identified, the mass was diagnosed as a primary acquired anterior perineal hernia, and laparoscopic repair was performed. A 2‐cm hernial orifice was found in the pouch of Douglas; the lumen extended toward the perineal region as confirmed by insertion of forceps into the orifice (Figure 3). The hernial orifice was closed by using nonabsorbable sutures, with additional closure of the uterosacral ligament with nonabsorbable sutures. The patient was discharged from the hospital 3 days postoperatively and has been symptom‐free for 8 months as of this writing.

## 3 DISCUSSION

Pelvic floor hernia is rare, and perineal hernia is an extremely rare pathologic state that surgeons might encounter once in their career,^2^ if ever. Stamatiou et al reported that Garangeot was the first to publish a case of primary perineal hernia in 1743.^3^ To our knowledge, Thomas first classified vaginal hernias into five groups in 1885.^4^ Furthermore, in 1940, Wilensky and Kaufman classified pelvic floor hernias as extravaginal, peritoneal vaginal, perineal, hedrocele, pudendal, and pelvic quasi hernias.^5^

Perineal hernia mainly occurs secondary. Many secondary perineal hernias occur after perineal gland operation within 1 year of the antecedent surgery.

Anterior perineal hernia occurs in the triangle‐shaped urogenital diaphragm surrounded by the ischiocavernosus, bulbocavernosus, and superficial transverse perineal muscles, and it affects only women.^2^ Because the anterior hernia is located in the urogenital diaphragm, the typical clinical symptom is prolapse around the labia. Critical cases advance to incontinence. For posterior perineal hernia, the typical symptom is a unilateral bulging in the gluteal or perineal region. In the present case, the patient had bulging of the labium major and was diagnosed with primary acquired anterior perineal hernia.

Clinical diagnosis of perineal hernia can be made using sonography, CT, MRI, and herniography^6^; an upright position during examination enables identification and anatomic association of the protruded bowel segment. Sometimes, perineal hernia may be mistaken for lipoma, fibroma, Bartholin cyst, rectocele, cystocele, or rectal prolapse. In our case, imaging showed no atrophy or pelvic floor muscular defect, and local external ultrasonography could visualize but not identify the hernia content.

Various approaches using mesh for closure of the pelvic defect have been reported for perineal hernia surgical repair, but the ideal approach has yet to be established. Sorelli et al recently reported THE advantages of the laparoscopic approach in perineal hernia repair.^7^ Ghellai et al and Franklin et al reported a laparoscopic approach using such mesh for pelvic defect closure.^8^, ^9^ For repair, direct suturing is considered an effective option, but it should be used with care because of its associations with relapse.^10^ Cali et al reported a recurrence‐free case using nonabsorbable mesh repair for a large pelvic floor defect.^1^ Simple approximation of the defect may be feasible in some cases, but in long‐standing cases, the pelvic floor is deficient and requires autologous or prosthetic materials. In our case, we chose laparoscopic surgery. We identified a hernial sac extending up to the labium from the pouch of Douglas. Because the hernial orifice was small and the patient was young and could possibly undergo surgery for uterine myoma or other pelvic surgery in the future, we opted to use a double suture closure method consisting of direct suturing plus uterosacral ligament suturing, instead of mesh. No sign of relapse has been noted for 8 months postoperatively.

Table 1 summarizes 29 retrievable case reports of primary perineal hernia with detailed descriptions, including our case. There were 4 congenital and 25 acquired cases. All six cases of anterior perineal hernia were in women; our case is the latest report of the anterior type. There were 23 cases of posterior perineal hernia. All the congenital cases were of posterior type. Most cases initially manifested with bulging. Hernia content comprised intestine in most cases. Prolapse of the urinary bladder or greater omentum was noted in rare cases. Surgical approaches included transabdominal in 13 cases, transperineal in 2 cases, abdominoperineal in 2 cases, and laparoscopic in 5 cases; an additional two cases were managed by follow‐up observation without surgery. Hernia relapse was reported in one case of anterior perineal hernia treated via a transabdominal approach using suture closure.^10^ The relapse occurred 18 months postoperatively and was treated with repeat surgery via a perineal approach. Mesh was used in 10 cases and placed via an abdominal approach in most cases, except for one case in which a perineal approach was used.

**Table 1:** Reported perineal hernia (n = 29)

	Author	Reported year	Age (y)	Gender	Congenital or acquired	Position	Symptom	Hernia content	Surgical approach	Procedure
Case 1	Kondo	1936	26	Female	Ac	P	Bulging	Bowel	Abdominal	Suture
Case 2	Hirata	1947	40	Female	Ac	P	Bulging	Bowel	None	Conservative
Case 3	Amos	1951	40	Female	Ac	P	Bulging	Small bowel	Abdominal	Suture
Case 4	Richard and Ruben	1968	53	Male	Ac	P	Bulging	Sigmoid colon	Abdominal	Suture
Case 5	Thomford and Sherman	1969	66	Male	Ac	P	Rectal pain	Small bowel	None	Conservative
Case 6	Coussement et al	1978	0 (day 0)	Female	C	P	Bulging	Rectum	N/A	N/A
Case 7	Coussement et al	1978	0 (day 2)	N/A	C	P	Bulging	N/A	N/A	N/A
Case 8	Sato et al	1979	44	Female	Ac	A	Bulging + pain	N/A	Abdominal	Suture (recurrence)
Case 9	Vincent et al	1984	70	Female	Ac	P	Painful mass	Rectum	Abdominal	Suture + muscle flap
Case 10	Hubbard and Egelhoff	1989	0 (day 1)	Female	C	P	Bulging	Colon	N/A	N/A
Case 11	Edward et al	1990	68	Male	Ac	P	Bulging	Sigmoid colon + small bowel	N/A	N/A
Case 12	Rebecca et al	1992	64	Female	Ac	P	Constipation	Sigmoid colon	Abdominal	Mesh
Case 13	Ito et al	1994	67	Female	Ac	P	Bulging	N/A	Abdominal	Suture + broad ligament of uterus patch
Case 14	Padilla et al	1999	35	Female	Ac	A	Bulging	N/A	Perineal	Mesh
Case 15	Yagi et al	2000	47	Female	Ac	A	Bulging	N/A	Laparoscopy	Mesh
Case 16	Kuroki	2001	72	Female	Ac	A	Bulging	N/A	Abdominal	Suture + mesh
Case 17	Amano	2002	68	Female	Ac	P	Discomfort	Sigmoid colon	Abdominal	Suture
Case 18	Mara et al	2005	68	Female	Ac	P	Discomfort	Sigmoid colon	Abdominoperineal	Mesh
Case 19	Preiss et al	2006	75	Female	Ac	P	Back pain	Small bowel + omentum	Abdominal	Suture
Case 20	Dirk et al	2006	67	Female	Ac	P	Protrusion	Small bowel	Abdominal	Suture + mesh
Case 21	Jessica et al	2009	57	Female	Ac	P	Fullness+ pain	Small bowel	Laparoscopy	Suture
Case 22	Washiro et al	2010	81	Female	Ac	P	Discomfort	Small bowel	Abdominal	Uterus patch
Case 23	Sorelli et al	2011	45	Female	Ac	A	Discomfort	N/A	Laparoscopy	Mesh
Case 24	Dragan et al	2012	0 (day 0)	Female	C	P	Bulging	Rectum	Perineal	Suture
Case 25	Raghunath and Rajgopal	2013	42	Female	Ac	P	Bulging	Small bowel + colon + urinary bladder	Abdominoperineal	Suture + mesh
Case 26	Kurumboor and Palanisami	2013	63	Female	Ac	P	Bulging	Urinary bladder	Laparoscopy	Mesh
Case 27	Jorge and Juan	2017	71	Male	Ac	P	Bulging	Sigmoid colon + rectum	Abdominal	Mesh
Case 28	Mistry et al	2018	87	Female	Ac	P	None (incidental)	Small bowel + omentum	N/A	N/A
Case 29	Our case		49	Female	Ac	A	Bulging	Unknown	Laparoscopy	Suture

Abbreviations: A, anterior; Ac, acquired; C, congenital; N/A, not available; P, posterior.

We reported this very rare case of primary acquired anterior perineal hernia as we believe it was a valuable clinical experience worth sharing.

## DISCLOSURE OF INTERESTS

The authors have no conflicts of interest to disclose and received no funding for this report.

## ETHICS STATEMENT

This study received institutional review board approval, and it conforms to the provisions of the Declaration of Helsinki. The subject gave informed consent, and patient anonymity was preserved.
